# (Cyanato-κ*N*){1-[(*E*)-phen­yl(pyridin-2-yl-κ*N*)methyl­idene]semicarbazidato-κ^2^ *N* ^1^,*O*}copper(II)

**DOI:** 10.1107/S1600536812035386

**Published:** 2012-08-23

**Authors:** Roji J. Kunnath, M.R. Prathapachandra Kurup, Seik Weng Ng

**Affiliations:** a Department of Applied Chemistry, Cochin University of Science and Technology, Kochi 682 022, India; b Department of Chemistry, University of Malaya, 50603 Kuala Lumpur, Malaysia; c Chemistry Department, King Abdulaziz University, PO Box 80203 Jeddah, Saudi Arabia

## Abstract

The Cu^II^ atom in the title compound, [Cu(C_13_H_11_N_4_O)(NCO)], is *N*,*N*′,*O*-chelated by the mono-deprotonated Schiff base ligand and it is also covalently bonded to the nitro­gen end of the cyanate ion. The Cu^II^ atom shows a square-planar coordination that is distorted towards square-pyramidal owing to an inter­molecular Cu⋯N_cyanate_ inter­action [2.623 (2) Å], which gives a centrosymmetric dimer. In the square-planar description, the Cu^II^ atom is displaced out of the square plane [r.m.s. deviation = 0.048 Å] by 0.084 (1) Å in the direction of the apical occupant. In the crystal, adjacent complex dimers are linked by an amine N—H⋯N hydrogen-bond pair, also giving a centrosymmetric cyclic association [graph set *R*_2_^2^(8)], generating a linear chain parallel to [110].

## Related literature

For the synthesis of the Schiff base, see: de Lima *et al.* (2008 ▶). For a related copper(II) derivative, see: Peŕez-Rebolledo *et al.* (2006 ▶). For graph-set notation, see: Etter *et al.* (1990 ▶).
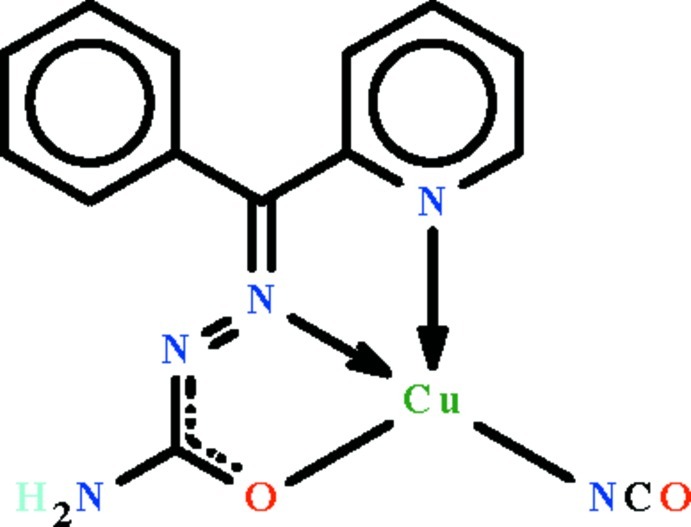


## Experimental

### 

#### Crystal data

[Cu(C_13_H_11_N_4_O)(NCO)]
*M* *_r_* = 344.82
Monoclinic, 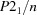

*a* = 8.7601 (1) Å
*b* = 7.6732 (1) Å
*c* = 20.0819 (3) Å
β = 96.7467 (7)°
*V* = 1340.52 (3) Å^3^
*Z* = 4
Mo *K*α radiation
μ = 1.64 mm^−1^
*T* = 293 K
0.30 × 0.25 × 0.20 mm

#### Data collection

Bruker Kappa APEXII diffractometer
Absorption correction: multi-scan (*SADABS*; Sheldrick, 1996 ▶) *T*_min_ = 0.638, *T*_max_ = 0.735
11886 measured reflections
3069 independent reflections
2728 reflections with *I* > 2σ(*I*)
*R*_int_ = 0.031

#### Refinement

*R*[*F* ^2^ > 2σ(*F* ^2^)] = 0.029
*wR*(*F* ^2^) = 0.093
*S* = 1.03
3069 reflections
208 parameters
2 restraints
H atoms treated by a mixture of independent and constrained refinement
Δρ_max_ = 0.37 e Å^−3^
Δρ_min_ = −0.47 e Å^−3^

### 

Data collection: *APEX2* (Bruker, 2010 ▶); cell refinement: *SAINT* (Bruker, 2010 ▶); data reduction: *SAINT*; program(s) used to solve structure: *SHELXS97* (Sheldrick, 2008 ▶); program(s) used to refine structure: *SHELXL97* (Sheldrick, 2008 ▶); molecular graphics: *X-SEED* (Barbour, 2001 ▶); software used to prepare material for publication: *publCIF* (Westrip, 2010 ▶).

## Supplementary Material

Crystal structure: contains datablock(s) global, I. DOI: 10.1107/S1600536812035386/zs2226sup1.cif

Structure factors: contains datablock(s) I. DOI: 10.1107/S1600536812035386/zs2226Isup2.hkl

Additional supplementary materials:  crystallographic information; 3D view; checkCIF report

## Figures and Tables

**Table 1:** Hydrogen-bond geometry (Å, °)

*D*—H⋯*A*	*D*—H	H⋯*A*	*D*⋯*A*	*D*—H⋯*A*
N4—H41⋯N3^i^	0.88 (1)	2.27 (1)	3.139 (2)	173 (3)

Symmetry code: (i) 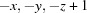.

**Fig. 1.**
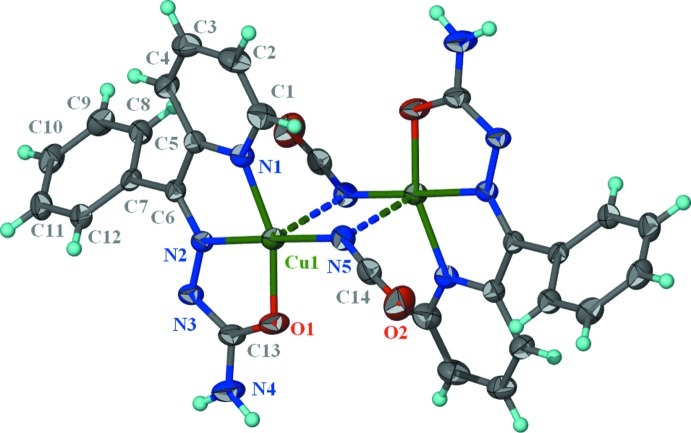
Thermal ellipsoid plot (Barbour, 2001) of two molecules of [Cu(NCO)(C13H11N4O)] that are linked by Cu···Ncyanate interactions (dashed bonds), at the 50% probability level. Hydrogen atoms are drawn as spheres of arbitrary radius.

## supplementary crystallographic information

### Comment

2-Benzoylpyridine semicarbazone (de Lima *et al.*, 2008) is a Schiff base that is capable of *N*,*N*',*O*-chelation to transition metal ions. This feature has been documented in a copper(II) dichloride adduct in which the Schiff base exists as a neutral molecule (Peŕez-Rebolledo *et al.*, 2006). However, the Cu^II^ atom in the title compound [Cu(NCO)(C_13_H_11_N_4_O)] (Scheme I) is *N*,*N*',*O*-chelated by the mono-deprotonated Schiff base ligand and it is also covalently bonded to the nitrogen end of the cyanate ion. The metal center shows square-planar coordination that is distorted towards square-pyramidal coordination owing to an intermolecular Cu···N_cyanate_ interaction [2.623 (2) Å], which generates a centrosymmetric dimer (Fig. 1). The geometry is better interpreted as square planar as the Cu···N_cyanate_···Cu angle is too acute [93.0 (1)°].

Adjacent dimers are linked by an amine N—H···N hydrogen-bond pair (Table 1), also giving a centrosymmetric cyclic association [graph set *R*^2^_2_(8) (Etter *et al.*, 1990], generating a linear chain parallel to [1 1 0].

### Experimental

A methanol solution (20 ml) of 2-benzoylpyridine semicarbazone (0.240 g, 1 mmol) (de Lima *et al.*, 2008), copper sulfate pentahydrate (0.249 g, 1 mmol) and sodium cyanate (0.065 g, 1 mmol) was heated for 5 h. The dark green solid was collected and recrystallized from methanol.

### Refinement

Carbon-bound H-atoms were placed in calculated positions (C—H = 0.93 Å) and were included in the refinement in the riding model approximation, with *U*_iso_(H) set to 1.2*U*_eq_(C). The amino H-atoms were located in a difference Fourier map, and were refined with a distance restraint of N—H = 0.88±0.01 Å and their displacement parameters refined. Only one H-atom is involved in the formation of a hydrogen bond.

### Crystal data

**Table:**

[Cu(C_13_H_11_N_4_O)(NCO)]	*F*(000) = 700
*M**_r_* = 344.82	*D*_x_ = 1.709 Mg m^−^^3^
Monoclinic, *P*2_1_/*n*	Mo *K*α radiation, λ = 0.71073 Å
Hall symbol: -P 2yn	Cell parameters from 838 reflections
*a* = 8.7601 (1) Å	θ = 2.4–28.3°
*b* = 7.6732 (1) Å	µ = 1.64 mm^−^^1^
*c* = 20.0819 (3) Å	*T* = 293 K
β = 96.7467 (7)°	Prism, dark green
*V* = 1340.52 (3) Å^3^	0.30 × 0.25 × 0.20 mm
*Z* = 4	

### Data collection

**Table:**

Bruker Kappa APEXII diffractometer	3069 independent reflections
Radiation source: fine-focus sealed tube	2728 reflections with *I* > 2σ(*I*)
Graphite monochromator	*R*_int_ = 0.031
ω scans	θ_max_ = 27.5°, θ_min_ = 2.7°
Absorption correction: multi-scan (*SADABS*; Sheldrick, 1996)	*h* = −10→11
*T*_min_ = 0.638, *T*_max_ = 0.735	*k* = −9→9
11886 measured reflections	*l* = −26→26

### Refinement

**Table:**

Refinement on *F*^2^	Secondary atom site location: difference Fourier map
Least-squares matrix: full	Hydrogen site location: inferred from neighbouring sites
*R*[*F*^2^ > 2σ(*F*^2^)] = 0.029	H atoms treated by a mixture of independent and constrained refinement
*wR*(*F*^2^) = 0.093	*w* = 1/[σ^2^(*F*_o_^2^) + (0.0579*P*)^2^ + 0.5121*P*] where *P* = (*F*_o_^2^ + 2*F*_c_^2^)/3
*S* = 1.03	(Δ/σ)_max_ = 0.001
3069 reflections	Δρ_max_ = 0.37 e Å^−^^3^
208 parameters	Δρ_min_ = −0.47 e Å^−^^3^
2 restraints	Extinction correction: *SHELXL97* (Sheldrick, 2008), Fc^*^=kFc[1+0.001xFc^2^λ^3^/sin(2θ)]^-1/4^
Primary atom site location: structure-invariant direct methods	Extinction coefficient: 0.0221 (17)

### Fractional atomic coordinates and isotropic or equivalent isotropic displacement parameters (Å^2^)

**Table:**

	*x*	*y*	*z*	*U*_iso_*/*U*_eq_	
Cu1	0.31070 (2)	0.50325 (3)	0.506177 (10)	0.03203 (12)	
O1	0.28257 (17)	0.3049 (2)	0.56285 (6)	0.0473 (4)	
O2	0.5831 (2)	0.6387 (3)	0.67349 (8)	0.0696 (6)	
N1	0.26412 (17)	0.6848 (2)	0.43675 (7)	0.0323 (3)	
N2	0.13987 (16)	0.38948 (19)	0.45304 (7)	0.0287 (3)	
N3	0.09476 (17)	0.2331 (2)	0.47511 (7)	0.0332 (3)	
N4	0.1503 (3)	0.0571 (3)	0.56674 (9)	0.0526 (5)	
H41	0.078 (2)	−0.018 (3)	0.5525 (15)	0.057 (8)*	
H42	0.196 (3)	0.034 (4)	0.6065 (8)	0.059 (8)*	
N5	0.47729 (18)	0.6213 (2)	0.56268 (8)	0.0391 (4)	
C1	0.3226 (2)	0.8437 (3)	0.43590 (10)	0.0425 (4)	
H1	0.3972	0.8773	0.4703	0.051*	
C2	0.2763 (3)	0.9611 (3)	0.38550 (13)	0.0480 (5)	
H2	0.3178	1.0727	0.3862	0.058*	
C3	0.1682 (2)	0.9104 (3)	0.33440 (11)	0.0448 (5)	
H3	0.1354	0.9875	0.2999	0.054*	
C4	0.1084 (2)	0.7441 (3)	0.33435 (9)	0.0383 (4)	
H4	0.0364	0.7071	0.2995	0.046*	
C5	0.15699 (19)	0.6327 (2)	0.38691 (8)	0.0299 (3)	
C6	0.09472 (19)	0.4563 (3)	0.39498 (8)	0.0289 (3)	
C7	−0.00451 (19)	0.3697 (2)	0.34034 (8)	0.0295 (3)	
C8	0.0435 (2)	0.3596 (3)	0.27703 (9)	0.0373 (4)	
H8	0.1360	0.4106	0.2691	0.045*	
C9	−0.0453 (2)	0.2744 (3)	0.22558 (9)	0.0438 (5)	
H9	−0.0119	0.2674	0.1834	0.053*	
C10	−0.1826 (2)	0.2003 (3)	0.23667 (10)	0.0453 (5)	
H10	−0.2419	0.1424	0.2021	0.054*	
C11	−0.2324 (2)	0.2114 (3)	0.29881 (11)	0.0438 (5)	
H11	−0.3263	0.1626	0.3060	0.053*	
C12	−0.1440 (2)	0.2947 (3)	0.35081 (9)	0.0351 (4)	
H12	−0.1779	0.3006	0.3929	0.042*	
C13	0.1784 (2)	0.2042 (3)	0.53522 (8)	0.0369 (4)	
C14	0.5253 (2)	0.6252 (3)	0.61718 (10)	0.0396 (4)	

### Atomic displacement parameters (Å^2^)

**Table:**

	*U*^11^	*U*^22^	*U*^33^	*U*^12^	*U*^13^	*U*^23^
Cu1	0.03601 (17)	0.03610 (18)	0.02273 (15)	−0.00921 (8)	−0.00184 (10)	−0.00184 (7)
O1	0.0603 (9)	0.0532 (9)	0.0253 (6)	−0.0211 (7)	−0.0086 (6)	0.0083 (6)
O2	0.0639 (10)	0.1105 (17)	0.0313 (7)	−0.0030 (10)	−0.0079 (7)	−0.0060 (9)
N1	0.0361 (7)	0.0323 (8)	0.0282 (7)	−0.0055 (6)	0.0017 (6)	−0.0025 (6)
N2	0.0310 (7)	0.0321 (7)	0.0227 (6)	−0.0053 (6)	0.0020 (5)	−0.0012 (5)
N3	0.0399 (7)	0.0341 (8)	0.0250 (6)	−0.0089 (6)	0.0013 (6)	0.0017 (6)
N4	0.0713 (13)	0.0508 (11)	0.0329 (9)	−0.0212 (10)	−0.0060 (8)	0.0121 (8)
N5	0.0370 (8)	0.0480 (10)	0.0316 (8)	−0.0072 (7)	0.0011 (6)	−0.0007 (7)
C1	0.0503 (11)	0.0374 (10)	0.0396 (10)	−0.0124 (8)	0.0041 (8)	−0.0064 (8)
C2	0.0609 (13)	0.0314 (9)	0.0536 (13)	−0.0093 (10)	0.0149 (10)	0.0014 (9)
C3	0.0528 (11)	0.0375 (11)	0.0452 (11)	0.0039 (9)	0.0104 (9)	0.0092 (9)
C4	0.0400 (9)	0.0381 (10)	0.0358 (9)	0.0018 (8)	−0.0002 (7)	0.0037 (8)
C5	0.0295 (7)	0.0314 (9)	0.0288 (7)	−0.0007 (6)	0.0034 (6)	−0.0017 (6)
C6	0.0270 (7)	0.0332 (8)	0.0261 (8)	−0.0013 (7)	0.0014 (6)	−0.0013 (7)
C7	0.0296 (7)	0.0297 (8)	0.0273 (7)	−0.0003 (6)	−0.0041 (6)	0.0010 (6)
C8	0.0344 (9)	0.0473 (11)	0.0295 (8)	−0.0053 (8)	0.0008 (7)	−0.0012 (8)
C9	0.0500 (11)	0.0512 (12)	0.0285 (8)	0.0002 (9)	−0.0031 (8)	−0.0051 (8)
C10	0.0465 (11)	0.0454 (12)	0.0396 (10)	−0.0040 (9)	−0.0142 (8)	−0.0070 (8)
C11	0.0317 (9)	0.0455 (11)	0.0515 (11)	−0.0083 (8)	−0.0062 (8)	0.0002 (9)
C12	0.0309 (8)	0.0368 (10)	0.0368 (9)	−0.0022 (7)	0.0013 (7)	0.0002 (7)
C13	0.0456 (10)	0.0403 (10)	0.0247 (8)	−0.0078 (8)	0.0039 (7)	0.0023 (7)
C14	0.0350 (9)	0.0442 (11)	0.0398 (10)	−0.0061 (8)	0.0060 (7)	−0.0032 (8)

### Geometric parameters (Å, º)

**Table:**

Cu1—O1	1.9335 (14)	C2—H2	0.9300
Cu1—N2	1.9404 (14)	C3—C4	1.379 (3)
Cu1—N5	1.9618 (16)	C3—H3	0.9300
Cu1—N1	1.9790 (15)	C4—C5	1.385 (2)
Cu1—N5^i^	2.6225 (17)	C4—H4	0.9300
O1—C13	1.272 (2)	C5—C6	1.475 (2)
O2—C14	1.188 (2)	C6—C7	1.475 (2)
N1—C1	1.324 (2)	C7—C8	1.388 (2)
N1—C5	1.349 (2)	C7—C12	1.388 (2)
N2—C6	1.293 (2)	C8—C9	1.382 (3)
N2—N3	1.354 (2)	C8—H8	0.9300
N3—C13	1.355 (2)	C9—C10	1.372 (3)
N4—C13	1.331 (3)	C9—H9	0.9300
N4—H41	0.876 (10)	C10—C11	1.372 (3)
N4—H42	0.869 (10)	C10—H10	0.9300
N5—C14	1.126 (2)	C11—C12	1.382 (3)
C1—C2	1.380 (3)	C11—H11	0.9300
C1—H1	0.9300	C12—H12	0.9300
C2—C3	1.368 (3)		
			
O1—Cu1—N2	79.99 (6)	C3—C4—C5	119.16 (18)
O1—Cu1—N5	99.25 (6)	C3—C4—H4	120.4
N2—Cu1—N5	177.52 (6)	C5—C4—H4	120.4
O1—Cu1—N1	159.83 (6)	N1—C5—C4	120.48 (17)
N2—Cu1—N1	81.22 (6)	N1—C5—C6	115.08 (15)
N5—Cu1—N1	99.23 (7)	C4—C5—C6	124.40 (16)
O1—Cu1—N5^i^	99.82 (6)	N2—C6—C7	125.67 (17)
N2—Cu1—N5^i^	95.41 (6)	N2—C6—C5	112.66 (15)
N5—Cu1—N5^i^	87.05 (6)	C7—C6—C5	121.65 (15)
N1—Cu1—N5^i^	89.15 (6)	C8—C7—C12	118.85 (16)
C13—O1—Cu1	110.84 (11)	C8—C7—C6	119.40 (16)
C1—N1—C5	119.97 (16)	C12—C7—C6	121.74 (16)
C1—N1—Cu1	127.54 (13)	C9—C8—C7	120.43 (18)
C5—N1—Cu1	112.47 (12)	C9—C8—H8	119.8
C6—N2—N3	125.23 (14)	C7—C8—H8	119.8
C6—N2—Cu1	116.88 (12)	C10—C9—C8	120.13 (19)
N3—N2—Cu1	117.03 (10)	C10—C9—H9	119.9
N2—N3—C13	106.76 (14)	C8—C9—H9	119.9
C13—N4—H41	124 (2)	C9—C10—C11	120.02 (17)
C13—N4—H42	121 (2)	C9—C10—H10	120.0
H41—N4—H42	114 (3)	C11—C10—H10	120.0
C14—N5—Cu1	137.89 (16)	C10—C11—C12	120.38 (18)
N1—C1—C2	121.99 (19)	C10—C11—H11	119.8
N1—C1—H1	119.0	C12—C11—H11	119.8
C2—C1—H1	119.0	C11—C12—C7	120.18 (18)
C3—C2—C1	118.8 (2)	C11—C12—H12	119.9
C3—C2—H2	120.6	C7—C12—H12	119.9
C1—C2—H2	120.6	O1—C13—N4	118.09 (17)
C2—C3—C4	119.53 (19)	O1—C13—N3	125.08 (17)
C2—C3—H3	120.2	N4—C13—N3	116.82 (17)
C4—C3—H3	120.2	N5—C14—O2	175.1 (2)
			
N2—Cu1—O1—C13	3.55 (14)	Cu1—N1—C5—C4	179.09 (14)
N5—Cu1—O1—C13	−178.85 (14)	C1—N1—C5—C6	−177.08 (16)
N1—Cu1—O1—C13	25.1 (3)	Cu1—N1—C5—C6	1.45 (19)
N5^i^—Cu1—O1—C13	−90.27 (14)	C3—C4—C5—N1	−1.5 (3)
O1—Cu1—N1—C1	150.16 (19)	C3—C4—C5—C6	175.87 (18)
N2—Cu1—N1—C1	171.58 (18)	N3—N2—C6—C7	−4.9 (3)
N5—Cu1—N1—C1	−5.94 (18)	Cu1—N2—C6—C7	164.10 (13)
N5^i^—Cu1—N1—C1	−92.81 (17)	N3—N2—C6—C5	176.72 (15)
O1—Cu1—N1—C5	−28.2 (3)	Cu1—N2—C6—C5	−14.28 (19)
N2—Cu1—N1—C5	−6.80 (12)	N1—C5—C6—N2	8.2 (2)
N5—Cu1—N1—C5	175.67 (12)	C4—C5—C6—N2	−169.37 (17)
N5^i^—Cu1—N1—C5	88.80 (12)	N1—C5—C6—C7	−170.28 (15)
O1—Cu1—N2—C6	−175.17 (14)	C4—C5—C6—C7	12.2 (3)
N1—Cu1—N2—C6	12.18 (13)	N2—C6—C7—C8	−126.9 (2)
N5^i^—Cu1—N2—C6	−76.12 (14)	C5—C6—C7—C8	51.4 (2)
O1—Cu1—N2—N3	−5.25 (12)	N2—C6—C7—C12	51.9 (3)
N1—Cu1—N2—N3	−177.90 (13)	C5—C6—C7—C12	−129.86 (18)
N5^i^—Cu1—N2—N3	93.80 (12)	C12—C7—C8—C9	−0.8 (3)
C6—N2—N3—C13	174.42 (17)	C6—C7—C8—C9	178.00 (19)
Cu1—N2—N3—C13	5.43 (18)	C7—C8—C9—C10	0.5 (3)
O1—Cu1—N5—C14	−22.4 (3)	C8—C9—C10—C11	0.4 (3)
N1—Cu1—N5—C14	149.4 (2)	C9—C10—C11—C12	−1.0 (3)
N5^i^—Cu1—N5—C14	−121.9 (3)	C10—C11—C12—C7	0.8 (3)
C5—N1—C1—C2	0.7 (3)	C8—C7—C12—C11	0.2 (3)
Cu1—N1—C1—C2	−177.57 (16)	C6—C7—C12—C11	−178.62 (18)
N1—C1—C2—C3	−1.0 (3)	Cu1—O1—C13—N4	176.97 (17)
C1—C2—C3—C4	0.0 (3)	Cu1—O1—C13—N3	−1.7 (3)
C2—C3—C4—C5	1.3 (3)	N2—N3—C13—O1	−2.4 (3)
C1—N1—C5—C4	0.6 (3)	N2—N3—C13—N4	178.93 (19)

Symmetry code: (i) −*x*+1, −*y*+1, −*z*+1.

### Hydrogen-bond geometry (Å, º)

**Table:**

*D*—H···*A*	*D*—H	H···*A*	*D*···*A*	*D*—H···*A*
N4—H41···N3^ii^	0.88 (1)	2.27 (1)	3.139 (2)	173 (3)

Symmetry code: (ii) −*x*, −*y*, −*z*+1.
